# Multidimensional Analysis of Disaster Nutrition: A Holistic Model Proposal Across Nutrition, Technology, Logistics, and Policy Axes

**DOI:** 10.3390/foods15010075

**Published:** 2025-12-26

**Authors:** Günay Basdogan, Osman Sagdic, Hakan Basdogan, Salih Karasu

**Affiliations:** 1 Department of Food Engineering, Faculty of Chemical and Metallurgical Engineering, Davutpasa Campüs, Yildiz Technical University, 34210 Istanbul, Turkey; gunaybasdogan@hotmail.com (G.B.); skarasu@yildiz.edu.tr (S.K.); 2 Erisler Food Industry and Trade Inc., 59850 Tekirdag, Turkey; hbasdogan@gmail.com

**Keywords:** disaster nutrition, integrated food system, food security, food logistics, sustainability

## Abstract

Over the past two decades, escalating climate crises, geopolitical conflicts, and pandemics have intensified the frequency and severity of disasters, exposing severe vulnerabilities in global food systems. In this pressing context, disaster nutrition emerges as a vital domain of intervention. However, existing academic literature and field practices often address this topic through fragmented, single-axis perspectives. Nutritional physiology, food technology, humanitarian logistics, and policy–ethics frameworks tend to progress in parallel yet disconnected tracks, which results in a lack of holistic models that adequately reflect field realities. The urgency of this issue is underscored by the latest global data. In 2023 alone, disasters resulted in over 86,000 deaths, a significant increase from the preceding two-decade annual average. Furthermore, the 2025 Global Report on Food Crises reveals that 295.3 million people faced high levels of acute food insecurity in 2024, marking the sixth consecutive year this number has risen. This escalating crisis highlights the inadequacy of fragmented approaches and necessitates the development of an integrated framework for disaster nutrition. To address this fragmentation, this study redefines disaster nutrition as a multi-layered, integrated food system challenge. Based on a comprehensive literature analysis, it proposes an “Integrated Disaster Food System Model” that brings these different dimensions together within a common framework. The model is built on four main components: (i) nutritional requirements and vulnerable groups (such as infants, older adults, pregnant individuals, and populations with chronic diseases requiring special diets); (ii) product design, technology, and packaging (balancing shelf life, nutritional value, cultural acceptability, and sensory attributes, including innovative components such as microalgae and fermented foods); (iii) logistics, storage, and distribution systems (centralized storage versus localized micro-warehouses, as well as the use of drones and digital traceability technologies); and (iv) policy, regulation, ethics, and sustainability (the applicability of the Sphere Standards, fair distribution, food waste, and environmental impact). By emphasizing the bidirectional and dynamic interactions among these components, the model demonstrates how decisions in one domain affect others (for example, how more durable packaging can increase both logistics costs and carbon footprint). The study highlights the risks and cultural mismatches associated with a “one-size-fits-all high-energy food” approach for vulnerable groups and argues for the necessity of localized, context-specific, and sustainable solutions. In conclusion, the article posits that the future of disaster food systems can only be shaped through a holistic approach in which interdisciplinary collaboration, technological innovation, and ethical–environmental principles are integrated into the core of policy-making.

## 1. Introduction

Over the past two decades, the climate crisis, biological outbreaks, geopolitical conflicts, and forced migration movements have markedly increased the frequency and severity of disasters [1]. This has rendered the global food system more fragile [1]. When natural disasters such as earthquakes, floods, droughts, hurricanes, and wildfires are compounded by protracted civil wars and mass displacement, the relationship between food insecurity and undernutrition becomes even more striking [1,2]. In disaster settings, the simultaneous disruption of production, storage, distribution, and consumption chains heightens the risk of both acute hunger and “hidden hunger” (micronutrient deficiencies). This situation, which leads to serious morbidity and mortality outcomes particularly for children, older adults, pregnant women, and individuals with chronic diseases, indicates that millions of people experience acute food insecurity [2,3,4]. Therefore, disaster nutrition needs to be positioned not merely as temporary support during and after a disaster, but as a strategic domain of intervention aimed at enhancing the resilience and capacity of structurally fragile food systems in the long term [1].

In early studies, disaster nutrition was approached through an energy-focused paradigm, relying primarily on relief food parcels, cereals, and canned foods [2]. The guidelines of organizations such as the World Health Organization (WHO) initially concentrated primarily on daily energy and protein requirements, while food diversity, cultural appropriateness, and special dietary needs remained secondary considerations [2,3]. One of the most important milestones in this field was the 2011 update of the Sphere Project, which, in addition to energy, defined population-level requirements for protein, fat, and a wide range of vitamins and minerals [3,4,5]. This development broadened the scope of the concept of “emergency food” in the contemporary literature. The term now encompasses not only products that supply calories, but also a broader category that includes “ready-to-use therapeutic foods (RUTF)” [6,7,8,9], as well as multidimensional criteria such as food safety, cultural acceptance, shelf life, functional properties, and logistical feasibility [4,5].

Despite this holistic framework, the current literature remains largely segregated along thematic axes [2,4,6,8,9,10,11]. One group of studies concentrates on nutritional physiology and clinical outcomes such as acute malnutrition [2,5,12], while another group focuses on food technology topics such as the formulation, nutritional value, and shelf life of emergency foods [5,9,10]. Research in humanitarian logistics and supply chain management addresses risks in food logistics [1,11,12,13], yet these findings are rarely integrated with the nutrition literature. Policy-oriented studies, on the other hand, discuss the field-level applicability of standards such as Sphere, but only partially integrate these standards with perspectives related to new food technologies or cultural adaptation [3,5,14]. Consequently, the axes of nutritional physiology, food technology, logistics, and policy often operate in parallel yet disconnected ways, and holistic models that adequately reflect field realities remain limited [2,6,11]. Addressing this interdisciplinary gap, this study poses the core research question: How can conflicting priorities in nutrition, technology, logistics, and policy be integrated within a holistic model that safeguards both operational efficiency and ethical principles?

This study aims to address this gap by examining disaster nutrition from an integrated food systems perspective and bringing these fragmented dimensions together within a single framework. To this end, the core and recent literature on the topic has been reviewed using a systematic approach. Through this literature review, the study seeks both to provide a conceptual framework for the scientific community and to offer a multidimensional reference model for the design of systems and products suited to disaster conditions for policymakers, practitioners, and the food industry. Finally, by detailing the current research gaps, it proposes focal areas and a research agenda for future studies.

### 1.1. Methodology

This study adopts a narrative review approach to analyze disaster nutrition through a multidimensional lens, integrating nutrition, food technology, logistics, and policy. A comprehensive literature search was conducted using the Web of Science, Scopus, PubMed, Dergipark and Google Scholar databases. The review covered peer-reviewed articles, international agency reports (e.g., WHO, WFP, UNHCR, Sphere Project), and policy documents published between 2000 and 2025 [14,15,16,17,18].

The search strategy employed the following keywords and their combinations: “disaster nutrition,” “emergency food systems,” “humanitarian logistics,” “food system resilience,” “ready-to-use therapeutic foods (RUTF),” and “sustainable food packaging.” The inclusion criteria focused on studies that addressed at least one of the four core axes (nutrition, technology, logistics, policy) within the context of natural or human-induced disasters. Articles were excluded if they focused solely on clinical medical treatments unrelated to food or lacked a systemic perspective.

The selected literature was subjected to thematic analysis to identify gaps in interdisciplinary integration. This analysis formed the basis for the proposed “Integrated Disaster Food System Model,” which aims to synthesize fragmented disciplinary knowledge into a cohesive framework.

### 1.2. Theoretical Background

A holistic examination of disaster nutrition from an integrated food systems perspective first requires a concise conceptualization of disaster management frameworks, nutritional standards, physiological responses, and food system resilience. The guidelines of organizations such as the World Health Organization (WHO), the World Food Programme (WFP), the United Nations High Commissioner for Refugees (UNHCR), and the International Federation of Red Cross and Red Crescent Societies (IFRC) generally define disaster management through the phases of preparedness–response–recovery–resilience, with the role of food differing across each phase [19,20,21]. During the response phase, the primary objective is to supply adequate energy, water, and essential micronutrients as quickly as possible; in the recovery phase, nutrition services are reorganized through local production and community-based solutions; in the resilience phase, emphasis shifts to strengthening the system’s ability to withstand and adapt to future shocks. Across these phases, disaster nutrition functions not merely as “caloric provision,” but as a pivotal policy instrument encompassing food safety, hygiene, cultural appropriateness, and sustainable supply mechanisms [3,18,19].

The Sphere Standards and related technical documents, which form the basis of these policies, define the daily per capita energy requirement under disaster conditions as approximately 2100 kcal, recommending that at least 10–12% of this energy come from protein and about 17–20% from fat [3,17,18]. Within the same framework, daily water needs are defined as a minimum of 15–20 L per person for drinking and basic hygiene [17,18]. By establishing minimum standards not only for energy and macronutrients but also for vitamins/minerals and food safety parameters, the Sphere framework provides a strong reference point for humanitarian assistance programs [3,17,18]. However, field data reveal significant challenges in meeting these standards: increased energy requirements in different climates, deviations from standard population assumptions among vulnerable groups (infants, pregnant individuals, older adults, people with chronic diseases), supply chain disruptions, local food preferences, and cultural/religious restrictions all hinder the full implementation of ration designs in real-world settings [3,4,18,21].

One of the main reasons for these field-level difficulties is the physiological response to crisis conditions, which becomes an equally significant determinant in disaster nutrition planning as logistics. Severe stress, trauma, infection burden, environmental changes, and variations in physical activity can increase basal metabolic rate and energy requirements, while also altering the need for protein and certain vitamins and minerals—particularly B-complex vitamins, vitamin C, vitamin A, iron, and zinc [2,18,22]. Vulnerable groups—children, pregnant and lactating women, older adults, and individuals with chronic diseases—are more susceptible in terms of immune function, thermoregulation, and digestion; thus disaster food formulations must provide additional energy and micronutrient support for these populations [10,18,21]. Studies conducted after recent earthquakes indicate widespread short-term weight loss, appetite loss, and dehydration in affected regions, findings associated with both calorie deficits and dietary/micronutrient insufficiencies [18,22].

Beyond individual physiology, disasters act as “stress tests” for the entire food system. In the food supply chain—comprising production, processing, storage, distribution, and consumption—the earliest vulnerabilities typically emerge in infrastructure and logistics (roads, bridges, cold chain, storage facilities), increasing risks related to stock management, expiry date control, and product losses [1,11,18].

At this point, the literature on food system resilience becomes particularly relevant. This concept refers to a food system’s capacity to maintain “adequate, appropriate, and accessible food” in the face of shocks, and is shaped by components such as robustness, flexibility, adaptation, and reorientation [23,24,25]. Local supply networks, short food chains, and community-based storage/distribution mechanisms are emphasized as elements that reduce dependency on external aid and strengthen recovery capacity in disaster situations [22,25,26]. Despite this, disaster food programs in many countries continue to rely on centralized, uniform, and import-dependent solutions—approaches that are neither desirable for food sovereignty nor supportive of long-term local economic recovery.

In addition to debates on system resilience, the sustainability and circular economy perspective has brought the environmental impacts of disaster foods to the forefront, challenging the traditional paradigm of “long shelf life and single-use packaging under all conditions” [27,28,29,30]. Reducing the carbon footprint is imperative, as food systems are major contributors to global emissions. Recent studies highlight that a significant portion of greenhouse gas (GHG) emissions originates directly from agricultural production systems, necessitating strict control mechanisms even in emergency food procurement [31,32,33]. Life cycle assessment (LCA) literature demonstrates that the linear “take–make–dispose” model in food and packaging systems is a major driver of greenhouse gas emissions, plastic pollution, and waste management crises In contrast, circular economy approaches highlight strategies such as reducing food waste, adopting reusable packaging, and developing menus based on local and seasonal production. In the disaster context, this perspective simultaneously acknowledges the need for high-energy, long shelf-life products, while promoting hybrid models that increase recycling options in packaging, reduce carbon intensity in logistics, and incorporate locally sourced ingredients into formulations. In this way, disaster nutrition can be framed not only as a matter of “survival” but also as part of a broader sustainability agenda aligned with long-term climate goals and circular economy principles [27,28,29,34].

Furthermore, to fully understand the complexities of these crises, disaster food systems should be conceptualized not merely as logistical operations but as “socio-technical systems.” In this view, technical elements (e.g., shelf-life technologies, transport drones) and social elements (e.g., dietary habits, policy regulations, vulnerable populations) are deeply intertwined. Consequently, failures in disaster nutrition often stem from “coupled system vulnerability,” where a disruption in one domain (e.g., a power outage affecting cold chains—a technical failure) triggers cascading effects in another (e.g., rejection of spoiled food leading to social unrest). Therefore, theoretical frameworks must move beyond simple “supply” logic to address these complex, non-linear interactions between social and technical components.

### 1.3. Global Perspective and Country-Level Case Applications

Different types of disasters significantly alter nutritional requirements from both physiological and logistical standpoints [3,18,22]. Sudden-onset disasters (earthquakes, hurricanes, flash floods) often collapse infrastructure within a short timeframe, prioritizing high-energy, ready-to-eat foods with minimal preparation and low water requirements during the first 72 h. In contrast, slow-onset disasters (droughts, global pandemics such as COVID-19, and prolonged conflicts) disrupt global supply chains and tend to produce long-term crises characterized by chronic energy and micronutrient deficiencies [3,18,19]. Urban disasters, due to high population density and vulnerable distribution infrastructures, require rapid supply mechanisms and large-scale shipments from outside the affected zone; rural disasters, however, lead to abrupt declines in local food self-sufficiency because of production losses and damaged transportation networks [1,14,24]. In human-induced disasters such as war, civil conflict, and mass displacement—as exemplified dramatically by the Syrian civil war and its resulting refugee crisis—food insecurity deepens not only through infrastructure destruction but also via economic collapse, price volatility, market disruptions, and heightened security risks [3,18,22]. Consequently, each disaster scenario demands distinct strategies with respect to food types and distribution models [1,14,24]. The devastating impact of such events was starkly illustrated in 2023, a year when earthquakes alone caused over 62,000 deaths and storms resulted in more than 14,000 fatalities globally (Table 1). Specific events, such as the earthquake in Türkiye that claimed over 50,000 lives and Storm Daniel in Libya which led to more than 12,000 deaths, exemplify the catastrophic scale of these sudden-onset emergencies and the immense logistical challenges they pose for nutritional intervention (Table 2).

The period of 2023–2024 emerges as a convergence point where natural disasters and human-induced crises have triggered each other, leading to an unprecedented global food and nutrition crisis. In 2023, natural disasters resulted in a total of 86,473 deaths, significantly above the twenty-year average (64,148), laying the groundwork for this crisis; this increase was primarily driven by severe earthquakes in Turkey and Syria (totaling 56,683 deaths) and intense storms such as the Daniel Storm in Libya (12,352 deaths), which caused extensive destruction (14,666 deaths) [34,35,36,37]. Beyond the loss of life, these disasters created multidimensional crises by disrupting food supply chains through destroyed infrastructure (roads, bridges, ports), collapsed logistics networks, and broken market systems, rendering even existing food physically and economically inaccessible [38]. As a direct consequence of this situation, combined with ongoing conflicts and economic shocks, by 2024, 295.3 million people in 53 countries faced high levels of acute food insecurity. The most concerning aspect of the crisis is that two million people—95% of whom are in Palestine (Gaza) and Sudan—faced the highest levels of famine risk in history (IPC/CH Phase 5), while 37.7 million children and 10.9 million pregnant/lactating women suffered from acute malnutrition. This complex picture underscores the vital importance of the “disaster nutrition” discipline and multi-faceted food solutions in emergency interventions; under these conditions, it is vital to provide not only calories but also long shelf-life, nutrient-dense foods tailored to the specific needs of different populations, such as ready-to-use therapeutic foods for children, special formulas for those with metabolic disorders like gluten intolerance, or easily swallowable nutritious purees for the elderly. The need for such strategic and inclusive nutrition solutions becomes even more urgent with an anticipated 45% decrease in humanitarian food funding in 2025, indicating a trajectory in which the ongoing crisis could deepen, placing millions of vulnerable people at even greater risk [34,35,36,37,38,39,40].

In response to these varied scenarios, international humanitarian organizations such as WFP, UNHCR, and IFRC employ standardized food packages tailored to different regions and disaster types [3,18,19]. Typical emergency food baskets distributed by these organizations include high-energy cereals, legumes, vegetable oil, and salt, while in rapid-onset emergencies, ready-to-eat meals, high-energy bars, and ready-to-use therapeutic foods (RUTF) for children are more commonly used [3,18,19]. The contents of these packages also vary by context: in long-term crises in Sub-Saharan Africa, WFP’s food basket typically relies on the cereal–legume–oil triad; in the Middle East, particularly in camps hosting Syrian refugees, distributions consist largely of culturally appropriate flour, rice, and canned foods [3,18,19]. Similarly, IFRC’s distributions in South Asian flood regions prioritize foods requiring no water for preparation and those that can be transported easily [3,18,19,41]. Yet even these standardized packages vary significantly depending on countries’ procurement capacity, logistical infrastructure, and the resilience of local markets after a disaster [1,14,21].

One of the most fundamental distinctions in disaster food strategies emerges from countries’ development levels and structural capacities [3,18,19]. In high-income countries, strategies are typically based on large stockpiles, strong supply chains, and diversified product ranges [3,18,19]. Following the 1995 Kobe earthquake and the 2011 Tōhoku earthquake–tsunami, Japan adopted a national policy of stocking long shelf-life ready foods (“kyūkan shokuhin”) in both municipal warehouses and households; ready-to-eat noodles, UHT beverages, and retort food packets constitute the core of this system [3,18,19]. In the United States, FEMA standardizes MRE (Meal, Ready-to-Eat) packages at federal and state levels, while the European Union has developed centralized crisis food reserves and shared stock management systems under the RescEU mechanism [3,18,19]. The common feature in these countries is that disaster food is regarded not only as humanitarian assistance but also as an integral component of national security and critical infrastructure strategy [3,18,19]. From the perspective of the proposed integrated model, these strategies exemplify a successful alignment of the “Policy” and “Technology” axes, where national regulations dictate the specific shelf-life technologies required for household stockpiling.

Conversely, structural and operational challenges are more pronounced in developing countries’ disaster nutrition practices [1,14,21]. Infrastructure limitations, insufficient cold-chain capacity, vulnerable transport networks, and inadequate storage facilities severely restrict post-disaster food supply [24]. The most concrete examples of these challenges were observed in Turkey during the 1999 Gölcük earthquake and especially the 2023 Kahramanmaraş earthquakes [25,26]. In these events, early-phase shipments from centralized warehouses led to logistical delays, reduced product diversity, and cultural mismatches in distributed foods [1,14,21]. This specific case illustrates a fundamental failure in the “Logistics” axis of the integrated model; although the production capacity existed, the collapse of the distribution network and lack of decentralized storage prevented the food from reaching the vulnerable groups effectively. However, in the following days, the deployment of mobile kitchens and the local availability of traditional durable foods—such as dried grains, fermented products, sun-dried vegetables, grain–legume blends, and yogurt-based dried foods—demonstrated the importance of localized and resilient solutions [1,14,21]. Despite their potential, the systematic integration of such local solutions into national disaster-food programs remains limited [1,14,21].

Taken together, these factors underscore the need for disaster nutrition strategies in developing countries to prioritize strengthening local production capacity, enhancing logistical resilience, and adopting culturally compatible, cost-effective shelf-life solutions [1,14,21].

## 2. Nutritional Requirements and Vulnerable Groups

Nutritional needs in disaster settings vary substantially depending on individuals’ physiological status, environmental conditions, and the duration of the crisis [1,3,14]. While the Sphere Standards establish an approximate requirement of 2100 kcal/day for the general adult population, this requirement may increase depending on the type of disaster, level of physical activity, hygiene conditions, and stress response [1,3,17]. The first 72 h constitute the “survival phase,” during which high-energy products that require no preparation and minimal water are essential; during this period, carbohydrate-based energy provision accompanied by minimal protein and fat support is recommended [1,13,15]. During the first week, continued metabolic stress raises energy requirements to 10–30% above basal levels, while protein needs increase significantly due to tissue repair and immune function demands [1,3,14]. In prolonged crises—such as refugee camps or extended post-earthquake/conflict environments—macro- and micronutrient needs must be sustainably met; deficiencies in B vitamins, vitamins A and D, iron, zinc, and iodine are particularly common [1,3,14]. Therefore, the formulation of disaster foods must consider not only caloric density but also vitamin–mineral content and bioavailability [1,9,19].

Beyond these general needs, infants, young children, older adults, and pregnant individuals constitute the most vulnerable groups during disasters and have unique nutritional requirements [36,37,38]. The sheer scale of this vulnerability is staggering; in 2024, an estimated 37.7 million children under five and nearly 10.9 million pregnant and breastfeeding women were suffering from acute malnutrition in food-crisis contexts [Table 3]. Of these children, over 10 million were classified as having severe acute malnutrition (SAM), a life-threatening condition. These statistics reveal a profound public health crisis that standard ‘one-size-fits-all’ food packages fail to address. Infants and children aged 0–24 months have a higher energy requirement per kilogram of body weight compared to adults, and appropriate texture, sterility, and allergen safety are essential [36,37,38]. Consequently, commercial infant formulas, age-appropriate complementary foods, and specialized micronutrient supplements must be included in disaster food packages for this group [38]. For pregnant and lactating women, daily energy requirements increase by approximately 300–500 kcal, with elevated needs for folate, iron, calcium, and omega-3 fatty acids [38]. Older adults may be unable to consume all foods included in standard packages due to chewing/swallowing difficulties, chronic diseases, or medication interactions; thus softer textures, low-sodium formulations, and easily digestible options are needed [39]. In these populations, disaster food design must consider functional accessibility (texture-modified foods), micronutrient density, and specialized medical foods, rather than focusing solely on energy content [40,41].

In addition to these groups, individuals with chronic diseases represent a frequently overlooked yet high-risk population in disaster nutrition planning [33,40]. For people with diabetes, high-glycemic emergency bars or sugar-containing ready meals may increase the risk of hyperglycemia; for individuals with hypertension, high-sodium canned foods and instant soups may cause severe cardiovascular complications [42,43]. The absence of gluten-free products in disaster food distributions can lead to acute gastrointestinal attacks and long-term malabsorption in individuals with celiac disease or gluten sensitivity [40,41,42]. For patients with kidney disease, standard high-protein or high-potassium rations may trigger acute exacerbations [44,45,46]. Literature indicates that disaster foods formulated specifically for chronic diseases remain extremely limited; in particular, low-sodium, low-sugar, gluten-free, and low-protein alternatives represent a major gap in both product design and logistics. This highlights the significant adverse risks posed by the “one-size-fits-all high-energy food” approach for vulnerable groups [43,44,45].

In addition to special dietary needs, allergen management is a crucial public health concern, especially in mass distribution settings. Common components of disaster food packages—such as dairy products, peanuts, soy, wheat/gluten, eggs, fish, and sesame—are among the most frequently reported food allergens. Standard emergency packages often contain multi-ingredient cereal blends or processed foods, increasing the risk of cross-contamination [45,46]. Therefore, allergen content must be clearly and simply labeled in multiple languages, supported with visual icons or color-coded warnings, and allergen-free alternative packages must be separated during mass distribution. Additionally, using the same containers, surfaces, or transport units in improvised kitchens or communal feeding sites without adequate cleaning increases cross-contamination risks, posing severe hazards particularly for children and individuals at risk of anaphylaxis. Thus, allergen management in disaster nutrition must be addressed holistically—not only at the product level but also throughout operational processes [46,47,48].

**Table 3:** Global Food Crises, Disaster-Related Food Insecurity and Nutrition Statistics: 2023–2025.

Category/Indicator	Key Figures	Year/Period
Overall Food Insecurity	295.3M people (22.6%) faced high acute food insecurity in 53 countries/regions. +13.7M increase from previous year.	2024/2023–2024
Crisis Severity (IPC/CH Phases)	~2M people in Catastrophe (Phase 5) in 5 countries; 95% in Palestine (Gaza) & Sudan. Sudan: 755,300 (0 in 2023). 36 countries: 35.1M in Emergency (Phase 4).	2024/2023–2024/Mar–Apr 2024
Undernutrition (Malnutrition)	37.7M children (6–59 mo) acutely malnourished in 26 countries. >10M SAM cases. 10.9M pregnant/lactating women affected in 21 countries. Worst: Sudan, Gaza, Yemen, Mali.	2024
Main Drivers	Conflict/Insecurity: 139.8M (20 countries)	2024
	Extreme Weather: 96.1M (18 countries)	2024
	Economic Shocks: 59.4M (15 countries)	2024
Forced Displacement	95.8M people displaced in 53 crisis countries	2024
Outlook & Financing	Humanitarian food funding may drop up to 45%; 14M children at risk of losing nutrition services	2025

Source: FSIN and GNAFC [44].

## 3. Innovative Food Development for Disaster Conditions

In disaster settings, the need for high-energy, nutrient-dense products that require minimal preparation has accelerated the development of innovative solutions in food technology. High-energy and highly nutritious products play a instrumental role, particularly during the first 72 h and the first week of a disaster. Among these products, energy bars, meal-replacement formulations, and RUTF/RUSF derivatives occupy a central position. RUTF-type products typically consist of peanut paste, milk protein, vegetable oil, sugar, and vitamin–mineral premixes; however, in recent years, research has increasingly focused on alternative plant-based proteins (pea, lentil, soy), cereal–legume blends, and formulations with lower allergenic potential as substitutes for milk protein [9,32,49,50,51,52]. In energy bar formulations, complex carbohydrates (such as oats and rice), healthy fats (canola, sunflower, coconut oil), and highly bioavailable protein sources are combined to create product profiles that provide both long shelf life and rapid recovery support. These products not only meet acute energy needs but are also enriched to help prevent micronutrient deficiencies in prolonged crises [52,53]. Despite their life-saving potential, RUTF products face criticism in the recent literature regarding “monotony” (flavor fatigue) and lack of cultural integration. Long-term reliance on imported RUTFs can hinder the recovery of local markets and often fails to address the diverse palate preferences of different populations, leading to food waste in prolonged crises.

New protein sources hold significant transformative potential for the future of disaster foods. Microalgae—particularly Spirulina and Chlorella species—are attractive ingredients for disaster food formulations due to their high levels of protein, essential amino acids, antioxidants, and pigments. Studies on microalgae-based bars, powdered drink mixes, biscuits, and soup bases indicate that these ingredients enhance both nutrient density and immune-supporting capacity [54,55]. Insect proteins are likewise proposed as strong alternatives for emergency rations, especially because of their highly bioavailable protein and fat content; however, they face substantial constraints in terms of halal/tayyib and kosher compliance, as well as cultural acceptability [56]. Consequently, insect-protein products largely remain at the pilot implementation stage in Europe and some Asian countries. Fermented foods also contribute significantly to disaster food technologies by simultaneously extending shelf life and improving digestibility. Fermented cereal–bar blends, yogurt powder–based mixes, and low-moisture prototypes fortified with stabilized probiotics represent emerging areas of research that combine durability with functional benefits [55,57].

Functional foods in disaster contexts are designed not only to provide energy but also to address broader health objectives such as immune modulation, stress management, support of cognitive functions, and mitigation of micronutrient deficiencies. Probiotic- and prebiotic-enriched products may offer protective effects against the elevated risk of gastrointestinal infections in mass shelter environments [57,58]. Fortification strategies implemented via vitamin–mineral-supplemented meal-replacement products—targeting deficiencies in iron, vitamin D, zinc, and vitamin B12—can yield clinically meaningful benefits in situations of long-term displacement [32,33]. In parallel, early-stage research on formulations containing adaptogens, nootropics, or herbal extracts is evaluating their potential effects on stress, sleep disturbances, and cognitive performance; however, it should be noted that such claims still lack a robust clinical evidence base in the specific context of disaster foods [58,59].

Cultural acceptability in product design is one of the most critical parameters determining the uptake of disaster foods. Standardized “one-size-fits-all food packages” distributed in many countries often exhibit low consumption rates when they do not align with the religious, cultural, and dietary habits of recipient communities, thereby contributing to clinically significant undernutrition. For this reason, expanding the availability of halal, kosher, vegetarian/vegan, and lactose-free/gluten-free options is essential [5,18,36,60]. Strategies to enhance cultural compatibility include cereal-based foods such as rice, bulgur, and bread-like products; noodles, couscous, and lentil-based soups; date-based bars; and spice blends tailored to local flavor preferences. Examples include ready-to-eat noodle packs widely used in Japan following tsunamis, rice–bulgur blends distributed in Middle Eastern refugee camps, and local cereal-based mixtures in African settings [28,31,54].

One of the forward-looking innovation areas is personalized disaster nutrition. Three-dimensional (3D) food printing technology offers a theoretical opportunity for disaster food design by enabling individualized textures, shapes, and nutrient profiles tailored to different age and dietary groups. Technically, it is possible to produce protein-enriched bars, soft-textured products for older adults, or fortified foods in playful shapes for children via 3D printing [59,60]. Nonetheless, these solutions remain at the laboratory scale, and their applicability in disaster zones is limited due to constraints related to energy requirements, equipment portability, cost, and raw material stability. That said, the literature has begun to discuss a future vision in which 3D printing technologies are deployed in humanitarian warehouses or regional production centers to develop long shelf-life, personalized products for disaster contexts.

## 4. Logistics, Distribution, and Emergency Food Systems

In disaster conditions, ensuring food supply depends not only on product formulation but also on the establishment of a robust and flexible supply chain. Supply chain vulnerability is particularly affected by factors such as road closures, damage to bridges and infrastructure, power outages, fuel shortages, and disruption of the cold chain. In earthquakes, floods, and humanitarian crises, one of the first challenges observed is the interruption of transport networks and the inability to deliver food stocks stored in central warehouses to the affected areas in a timely manner. For this reason, in recent years, many international organizations have emphasized that, alongside centralized storage models, decentralized regional micro-warehouse structures offer greater resilience. Localized (decentralized) production and storage systems reduce the risk of “single-point dependency” and enable faster response to disaster areas [61,62,63]. This logistical challenge is amplified by the massive scale of human displacement; in 2024, 95.8 million forcibly displaced people, including refugees and internally displaced persons (IDPs), were located within food-crisis contexts (Table 3). Reaching these mobile and often difficult-to-access populations is a primary obstacle that necessitates decentralized logistics and innovative distribution models, such as the micro-warehouses and drone delivery systems discussed herein.

The models used for emergency food distribution also vary depending on the geographical characteristics of the affected area, infrastructure capacity, and the size of the target population. Conventional convoy- and truck-based distribution systems remain the most common approach; however, in flood, landslide, or conflict zones, these methods are often constrained. Among alternative distribution methods, mobile kitchens and field kitchens play a particularly important role. These systems make it possible to prepare hot meals in mass shelter settings, thereby increasing food diversity and meeting communal feeding needs. In recent years, drones, unmanned ground vehicles, and autonomous marine vehicles have been increasingly employed to deliver small-volume yet high-value food items (e.g., medical nutrition products, infant formula, gluten-free foods) to critical locations. Drone-based distribution models provide effective solutions especially in mountainous regions and disaster scenarios where access is severely limited [64,65]. However, these systems are still constrained by weather conditions, payload capacity, and energy requirements.

Digital tools represent a transformative turning point in the modernization of disaster food logistics. Geographic Information Systems (GIS)-based spatial analyses map the distribution of affected populations, damage intensity, and regional food needs, enabling more efficient planning of distribution operations. Such models offer high accuracy for warehouse siting, route optimization, and transport risk assessments. Digital platforms used in disaster management allow real-time stock monitoring, demand forecasting, and evaluation of supply capacity. Blockchain-based systems are increasingly proposed, particularly for enhancing the traceability of donation-based food flows, food safety, and fraud prevention; by recording every movement in the supply chain in a verifiable manner, this technology can improve the reliability of disaster food distributions [66,67,68].

The environmental impacts of logistics systems have also become an important topic of debate in the disaster management literature. Green logistics approaches aim to reduce the carbon footprint of transport modes, increase energy efficiency, and promote the development of sustainable vehicle infrastructure. Since the majority of shipments to disaster zones are carried out with diesel-powered vehicles, associated emissions constitute a significant concern. As a result, multimodal logistics (road–rail–sea combinations) is increasingly considered from both cost and environmental perspectives. The use of solar energy in warehouses, low-consumption cold storage systems, electric or hybrid transport vehicles, green warehouse certifications, and energy management software are among the solutions proposed for disaster logistics. In addition, short supply chain models (local sourcing) not only reduce the carbon footprint but also support economic recovery in the aftermath of disasters [69,70,71].

## 5. Policies, Regulations, and Ethics

International policy and standard frameworks play a guiding role—both operationally and ethically—in shaping disaster food systems [43,44,45,46]. The Sphere Standards, Codex Alimentarius, and the guidance documents of United Nations agencies (WHO, WFP, UNHCR) establish minimum requirements related to nutrition, food safety, water sanitation, and logistics management [43,44,45,46]. Within this context, Sphere’s provisions on energy and macro/micronutrient needs, the prioritization of foods requiring minimal preparation, access to safe water, and culturally acceptable products are of vital importance [46]. However, field observations and evaluation reports indicate that many countries struggle to meet these standards fully due to the nature of disasters, supply chain disruptions, logistical constraints, and cost pressures [72,73,74]. The rapid procurement of low-quality, inappropriate, or culturally incompatible foods during emergencies further highlights the gap between international standards and practical implementation [75,76,77].

The national-level reflection of these international frameworks also shapes the foundational structures of disaster food systems [78,79,80,81,82]. FEMA in the United States provides an advanced model through strategic food stockpiles and long-shelf-life “MRE” (Meals Ready-to-Eat) products, while the European Union has established common crisis food reserves and standardized frameworks under shared civil protection mechanisms [83,84,85]. In Turkey, AFAD’s strategic storage policies, nutritional regulations in mass shelter settings, and coordination mechanisms are becoming more institutionalized [86,87,88,89]. Despite these improvements, the literature indicates that significant policy gaps persist in many countries regarding supply continuity, funding allocation, integration with local producers, and the standardization of shelf life–nutritional value balances [90,91,92,93].

Where policy and standards fall short, the ethical dimension of disaster nutrition becomes especially prominent [88,89]. This dimension is not limited to equitable and inclusive food distribution; it also encompasses the protection of cultural, religious, and societal sensitivities [90,91,92]. Priority-setting in aid distribution, neglect of vulnerable groups (infants, older adults, individuals with disabilities or chronic illnesses), lack of transparency at distribution points, and accountability issues constitute the most critical ethical tensions observed in the field [93,94,95]. Cultural and religious incompatibility—for example, distribution of non-halal or non-kosher food products to Muslim or Jewish communities—not only undermines public trust but also reduces the effectiveness of aid programs [43,44,45,46]. Furthermore, growing criticism in the literature centers on “the commercialization of disasters,” referring to unethical practices such as turning disaster foods into market opportunities and price speculation [96]. These concerns underscore that ethical principles must lie at the core of operational guidelines as well as policy and oversight mechanisms in emergency food systems [97,98].

In addition to existing policy and ethical debates, regulatory gaps emerging from new technologies present another major challenge [98,99,100]. Innovative solutions such as microalgae, insect proteins, microbial fermentation products, and biodegradable packaging are not yet regulated in many countries within the disaster food context [101,102,103]. Therefore, emergency situations require accelerated approval pathways, rapid risk assessment mechanisms, and the integration of cultural/religious suitability criteria [104]. A lack of international harmonization persists, particularly concerning the application of halal, kosher, or sustainability labels under disaster conditions [95,101]. Frequently emphasized needs in the literature include adapting food safety legislation to emergency contexts, integrating rapid testing methods, defining environmental impact criteria for packaging materials, and accelerating the safety assessment of next-generation ingredients [105,106,107].

## 6. Discussion: The Integrated Disaster Food System Model

A multidimensional synthesis of the disaster nutrition literature reveals that findings from different disciplines often emphasize priorities that may conflict with one another. Research on nutritional requirements and vulnerable groups highlights the vital importance of providing high-energy and essential micronutrient support during the first 72 h and the first week of a disaster. However, sensory acceptability, cultural compatibility, and special dietary needs are frequently deprioritized. Conversely, consumer studies and field observations report that some high–energy-dense products (e.g., RUTF-like bars) show low acceptance and, when consumed over extended periods, may increase the risk of undernutrition due to appetite loss, gastrointestinal discomfort, or monotonous diets [14,108,109].

Meanwhile, technology- and shelf-life–oriented studies show that durability-enhancing thermal or alternative processing techniques increase costs, reduce sensory quality, or generate greater packaging waste. Logistic research identifies durable, lightweight, long-shelf-life products as operationally ideal, but these attributes do not always align with nutritional quality, sustainability, or cultural acceptability [15,104,110]. Policy and ethical perspectives further complicate the picture: although reducing product diversity may seem practical to enable rapid distribution, standardized packages that fail to address the needs of vulnerable groups introduce both ethical and operational challenges. Collectively, these findings indicate that one-dimensional approaches are inadequate, and that holistic models incorporating food safety, nutritional value, acceptability, cost, logistics, and ethics are increasingly necessary [84,85,86,87]. The human cost of failure is severe/catastrophic: in 2024, approximately 2 million people endured ‘Catastrophe’ (IPC Phase 5) levels of food insecurity, a state of extreme starvation. Compounding this, humanitarian funding for food sectors is projected to fall by as much as 45% in 2025, placing millions more at risk (Table 3).

**Figure 1:**
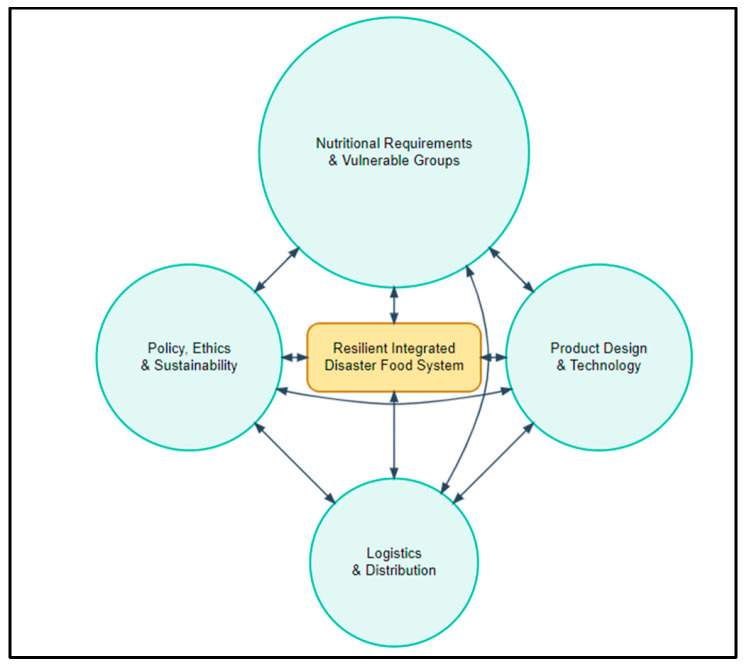
The Integrated Disaster Food System Model. The diagram illustrates the four core components (Nutrition, Technology, Logistics, Policy) and their bidirectional interactions, demonstrating how decisions in one axis influence the resilience of the entire.

Within this framework, the Integrated Disaster Food System Model proposed in this study brings together nutrition, food technology, supply chain dynamics, policy, and ethical dimensions under a unified structure. The model is built upon four main components: (i) nutritional requirements and vulnerable groups, (ii) product design, technologies, and packaging, (iii) logistics, storage, and distribution systems, and (iv) policy, regulations, ethics, and sustainability [110,111,112,113,114]. As illustrated in Figure 1, the proposed model visualizes the bidirectional dependencies and feedback loops between these four components. It demonstrates that these elements are not independent; rather, they interact dynamically.

These components are not independent; rather, they interact bi-directionally and influence one another. For example, the technology used in product design directly affects logistics costs (weight, volume, required storage temperature) as well as policy compliance (packaging regulations, cultural suitability, food safety requirements). Likewise, defining nutritional requirements necessitates the development of products tailored for vulnerable groups, yet these products must also meet stability and cost thresholds to remain logistically viable for large-scale distribution. Policies and ethical principles function as an overarching layer that shapes every component—issues such as the acceptance of new protein sources, criteria for green logistics, cultural compatibility, equitable distribution, and transparency form the fundamental framework for a functional system. In the model diagram, bidirectional arrows among components illustrate how improvements in one part of the system may unintentionally compromise another, or alternatively, generate reinforcing positive effects [88,89,90].

Based on this integrated framework, several recommendations emerge for policy and practice.

First, during the preparedness phase, countries must not only maintain strategic food reserves but also develop validated product portfolios tailored to different demographic groups. These portfolios should include dedicated emergency food sets for infants, older adults, individuals with celiac disease, diabetics, and specific ethnic/religious communities.

Second, strengthening localized production networks, short supply chains, and regional micro-warehouse models may enhance both the speed and efficiency of post-disaster distribution. Public–private–academic collaborations are critical for innovating product design, durability testing, sustainable packaging solutions, and supply chain optimization.

Third, community preparedness—through household-level stocking of durable foods, digital tracking system implementation, and regular supply chain drills—can significantly reinforce operational capacity.

Finally, systematically integrating cultural compatibility, environmental sustainability, and ethical principles into policy documents ensures that disaster food systems support not only survival but also healthy and dignified nutrition.

These recommendations collectively give shape to the growing call in the literature for the transformation of disaster food systems toward holistic, multi-layered, and resilient models [91,92,93,94].

## 7. Future Research Directions

Although research on disaster foods has expanded considerably in recent years, significant gaps persist—particularly in areas such as sustainability, innovative ingredients, digital traceability, and artificial intelligence–based decision-support systems. As such, new-generation research programs grounded in interdisciplinary approaches are crucial both for advancing the scientific literature and for informing policy design and practical implementation.

Environmental impact assessment of disaster foods remains relatively underexplored. Life Cycle Assessment (LCA) is a suitable methodology for comprehensively analyzing the effects of energy-intensive processes (freeze-drying, UHT, retort processing), multilayer packaging, and long-distance logistics. The lack of comparative LCA studies evaluating different production techniques, packaging options, and distribution modes poses a major barrier to designing sustainable disaster foods. Future research must develop scenario models that incorporate environmental indicators such as carbon footprint, water footprint, packaging waste, recycling potential, and green logistics integration. Additionally, evaluating the environmental performance of localized production and decentralized logistics models is essential for aligning national disaster strategies with low-carbon food system transitions [95,96,97].

Among next-generation protein sources, microalgae, insect proteins, and microbial fermentation products are viewed as strong candidates for disaster food formulations due to their high nutrient density and environmental sustainability advantages. However, field-based pilot studies on these products remain extremely limited. Microalgae-based bars, high-protein fermented foods, and insect-based emergency rations must be evaluated for sensory acceptability as well as cultural and religious compatibility. The halal/tayyib framework, in particular, is a decisive factor in acceptance among sensitive populations; thus, assessing new-generation ingredients in terms of safety, process compliance, and community perception within this framework is necessary. Moreover, further research is needed on the storage stability of microalgae, post-processing nutrient losses, oxidative resistance, and antimicrobial properties to determine their suitability for disaster food systems [98,100,101].

Digital traceability and smart packaging systems also represent promising future directions. IoT-enabled active packaging technologies, equipped with sensors that monitor temperature, humidity, oxygen levels, or shelf-life indicators in real time, offer significant advantages for both safety and logistics planning. Integrating blockchain into supply chains can enhance transparency between donors, institutions, and disaster-affected populations, thereby increasing trust and accountability. However, practical implementation of such digital tools in the field remains insufficiently examined, particularly with regard to energy requirements, cost-effectiveness, and infrastructure dependency. Future studies should focus on low-energy sensors, offline-compatible blockchain architectures, and disaster-resilient data management systems [102,103]. Crucially, future research should not only develop these technologies but specifically test their integration within the proposed model. For example, studies should evaluate whether the implementation of smart packaging (Technology) significantly reduces transportation losses (Logistics) in real-world scenarios, or if the energy requirements render it unfeasible under current sustainability policies.

Finally, artificial intelligence and decision-support systems represent a highly promising yet underutilized research avenue in disaster nutrition. Machine learning–based optimization models can significantly improve critical logistical processes such as resource allocation, route planning, storage capacity management, and demand forecasting. Similarly, AI can contribute to dynamic menu planning based on nutritional requirements, personalized emergency food kit design for vulnerable groups, and scenario simulations for supply chain planning. Future research should develop explainable AI (XAI)–based decision-support platforms that integrate multivariate data sources (climate patterns, demographic distribution, disaster intensity, health records, and stock data) to strengthen the resilience of disaster food systems. Real-time data flows, predictive modeling, and simulation-based policy testing are among the innovations expected to shape the future of disaster food management [104,105].

## 8. Conclusions

This study reconceptualizes disaster nutrition beyond a mere ‘emergency intervention,’ framing it as a multilayered food system challenge encompassing sustainability, technology, logistics, policy, and ethics. The literature review demonstrates that existing research often adopts a single-axis perspective—focusing solely on nutritional needs, food technology, or logistics management—which limits the development of holistic solutions required by real-world disaster contexts. Effective food intervention in disaster scenarios necessitates a delicate balance among nutritional value, cultural acceptability, safety, shelf life, logistical feasibility, and environmental sustainability. When this balance is not achieved, issues such as rejection of products in the field, unmet needs of vulnerable groups, increased logistical costs, or long-term health risks emerge.

Within this context, the study integrates nutritional requirements, product design and processing technologies, logistics and distribution systems, and policy–ethics–sustainability dimensions into a unified framework through the proposed Integrated Disaster Food System Model. The proposed model demonstrates that decisions within a disaster food system entail direct or indirect trade-offs across components—for instance, increasing packaging durability may enhance logistical efficiency but elevate the carbon footprint or formulation adjustments required for cultural compatibility may affect cost or shelf life. These multidirectional interactions highlight the need for holistic evaluation tools and interdisciplinary collaboration in disaster nutrition. The study also identifies gaps in field implementation, discrepancies between standards and practice, and persistent shortcomings in meeting the needs of vulnerable groups, thereby offering a guiding framework for future research and policy development.

In conclusion, the future of disaster food systems depends on advancements in sustainable production, smart packaging, AI-supported decision systems, and localized supply chains. This study provides a comprehensive conceptual contribution to the academic literature while also offering actionable strategic roadmaps for institutions, policymakers, and the food industry. In a world where the frequency and intensity of disasters are increasing, the development of resilient, inclusive, and sustainable disaster food systems is not only a technical requirement but also a humanitarian imperative. Strengthening interdisciplinary approaches, developing field-tested product portfolios, and integrating environmental and ethical principles into policy frameworks are, therefore, of critical scientific and societal importance.

## Figures and Tables

**Table 1:** Number of Deaths by Disaster Type: 2023 Compared to 2003–2022 Annual Average [34].

Disaster Type	Number of Deaths in 2023	2003–2022 Annual Average Number of Deaths
Drought	247	1157
Earthquake	62,451	35,124
Extreme temperature	406	11,470
Flood	7763	5518
Mass movement (dry)	0	35
Mass movement (wet)	654	803
Storm	14,666	10,017
Volcanic activity	23	80
Wildfire	264	86
Total	86,473	64,148

Source: CRED [34].

**Table 2:** Top 10 Mortality Countries in 2023.

Country	Disaster Type	Number of Deaths
Türkiye	Earthquake	50,783
Libya	Storm Daniel	12,352
Syrian Arab Rep.	Earthquake	5900
Congo (Democratic Rep.)	Flood	2970
Morocco	Earthquake	2946
Afghanistan	Earthquake	2445
India	Flood	1529
Malawi	Tropical Storm Freddy	1209
Nigeria	Flood	275
Yemen	Flood	248

Source: CRED [34,35,36].

## Data Availability

No new data were created or analyzed in this study. Data sharing is not applicable to this article.
